# Engineering Cofactor Specificity of a Thermostable Phosphite Dehydrogenase for a Highly **Efficient** and Robust NADPH Regeneration System

**DOI:** 10.3389/fbioe.2021.647176

**Published:** 2021-04-01

**Authors:** Gamal Nasser Abdel-Hady, Takeshi Ikeda, Takenori Ishida, Hisakage Funabashi, Akio Kuroda, Ryuichi Hirota

**Affiliations:** ^1^Department of Molecular Biotechnology, Graduate School of Advanced Sciences of Matter, Hiroshima University, Hiroshima, Japan; ^2^Department of Genetics, Faculty of Agriculture, Minia University, Minia, Egypt; ^3^Unit of Biotechnology, Division of Biological and Life Sciences, Graduate School of Integrated Sciences for Life, Hiroshima University, Hiroshima, Japan

**Keywords:** phosphite dehydrogenase, cofactor regeneration, thermotolerance, organic solvent tolerance, nicotinamide adenine dinucleotide phosphate

## Abstract

Nicotinamide adenine dinucleotide phosphate (NADP)-dependent dehydrogenases catalyze a range of chemical reactions useful for practical applications. However, their dependence on the costly cofactor, NAD(P)H remains a challenge which must be addressed. Here, we engineered a thermotolerant phosphite dehydrogenase from *Ralstonia* sp. 4506 (RsPtxD) by relaxing the cofactor specificity for a highly efficient and robust NADPH regeneration system. The five amino acid residues, Cys174–Pro178, located at the C-terminus of β7-strand region in the Rossmann-fold domain of RsPtxD, were changed by site-directed mutagenesis, resulting in four mutants with a significantly increased preference for NADP. The catalytic efficiency of mutant RsPtxD_HARRA_ for NADP (*K*_cat_/*K*_M_)^NADP^ was 44.1 μM^–1^ min^–1^, which was the highest among the previously reported phosphite dehydrogenases. Moreover, the RsPtxD_HARRA_ mutant exhibited high thermostability at 45°C for up to 6 h and high tolerance to organic solvents, when bound with NADP. We also demonstrated the applicability of RsPtxD_HARRA_ as an NADPH regeneration system in the coupled reaction of chiral conversion of 3-dehydroshikimate to shikimic acid by the thermophilic shikimate dehydrogenase of *Thermus thermophilus* HB8 at 45°C, which could not be supported by the parent RsPtxD enzyme. Therefore, the RsPtxD_HARRA_ mutant might be a promising alternative NADPH regeneration system for practical applications.

## Introduction

Biocatalysts play a crucial role in the chemical, pharmaceutical, and energy industries (Bornscheuer et al., 2012; Straathof, 2014; Wang et al., 2017). Over the last few decades, the use of enzymes in various biotechnology-related fields has drastically increased and is growing continuously (Wandrey et al., 2000; Schmid et al., 2001). Their catalytic performance in benign reactions along with high selectivity makes them valuable tools for sustainable industrial processing (Woodyer et al., 2006; Hollmann et al., 2010; Wang et al., 2017). Recent advances in genomics, metagenomics, and gene synthesis technology have significantly increased the number of enzymes and are being tailored as specific biocatalyst for bioproduction. However, approximately 65% of industrially used enzymes only catalyze simple hydrolytic reactions, and many useful reactions remain unused because of their dependency on costly cofactors (van der Donk and Zhao, 2003; Faber, 2004). In particular, dehydrogenases, a family of enzymes catalyzing a wide range of industrially useful reactions such as chiral conversions of pharmaceutical building blocks, require nicotinamide cofactors, NAD(H) or NADP(H), which precludes their large-scale use in stoichiometric amounts (Hummel and Kula, 1989; Krix et al., 1997).

To overcome such limitations, cofactors are regenerated *in situ* using cheaply available sacrificial substrates (Wichmann and Vasic-Racki, 2005). Implementation of such cofactor regeneration system can reduce production costs and increase productivity by preventing product inhibition and/or by promoting the main reaction owing to the thermodynamically strong driving force of the regeneration reaction (Chenault and Whitesides, 1987; Wu et al., 2013). Currently, several enzymes, including formate dehydrogenase (FDH) (Wichmann et al., 1981), alcohol dehydrogenase, glucose 6-phosphate dehydrogenase, and glucose dehydrogenase (GDH), have been reported to generate NADH (Wichmann and Vasic-Racki, 2005; Weckbecker et al., 2010). Among them, FDH from *Candida boidinii* is the most widely used enzyme and is shown to support the production of L-*tert*-leucine on a multi-ton scale (Tishkov and Popov, 2006). GDH and FDH are also used as a NADPH regeneration system. GDH is superior owing to its high specific activity and the low cost of glucose, its substrate. Meanwhile, FDH offers strongly driven reaction owing to its free-energy changes, and the by-product of its reaction, CO_2_, does not affect the reaction’s conditions. Although originally isolated FDHs from bacteria cannot accept NADP as a cofactor, a mutant FDH from *Pseudomonas* sp. 101 (mut-PseFDH) that utilized NADP was created and applied to NADPH regeneration for the synthesis of chiral (*R*)-alcohols and lactones (Seelbach et al., 1996; Rissom et al., 1997). However, both GDH and FDH have drawbacks; GDH produces gluconic acid as a by-product, inhibiting the main reactions due to pH changes during reactions (Tishkov and Popov, 2006). FDH exhibits lower catalytic efficiency than other regeneration enzymes (Tishkov et al., 1999). Consequently, owing to the expense of NADPH and the lack of a good regeneration system, a number of useful NADPH-dependent enzymes remain unemployed. Therefore, it is important to develop a highly active and economically relevant NADPH regenerative enzyme for bioindustrial manufacturing.

Phosphite dehydrogenase (PtxD), which catalyzes the oxidation of phosphite to phosphate concomitant with the reduction of NAD, was originally isolated from *Pseudomonas stutzeri* WM88 (PsePtxD) (Costas et al., 2001). Following this finding, PtxD was isolated from several bacteria inhabiting soils and aquatic environments (White and Metcalf, 2007; Hirota et al., 2012). PtxD has received considerable research attention as an alternative cofactor regeneration system due to several advantages: firstly, owing to the large change in free energy of this reaction (Δ*G*°’ = −63.3 kJ/mol), NADH regeneration by PtxD is more strongly driven than any other NADH regeneration enzyme; secondly, phosphate generated by phosphite oxidation can serve as a buffer for the main reaction (Vrtis et al., 2002; Relyea and van der Donk, 2005); and thirdly, phosphite is also available from the by-products of several industrial processes, thus ensuring an economically feasible reaction process (Kuroda and Hirota, 2015). Since heterologous expression of PtxD can expand the phosphorus substrate repertoire of various organisms, PtxD has also received an increased attention as a tool for selective cultivation (Kanda et al., 2014; Shaw et al., 2016) and biological containment of bacteria (Hirota et al., 2017; Motomura et al., 2018). However, naturally isolated PtxD enzymes, including PsePtxD, generally show low heat stability and prefer NAD as a substrate, hindering their application as a practical NADPH regeneration system. Johannes and coworkers created a PsePtxD mutant (12×) with a half-life at 45°C of approximately 7,000-fold higher than that of the wild-type enzyme (Johannes et al., 2005). Meanwhile, Woodyer et al. (2003) successfully altered its cofactor specificity by creating a mutant PsePtxD with two amino acids replacements at the NAD-binding site in the Rossmann-fold domain (PsePtxD_E__175__A/A__176__R_). However, the simultaneous introduction of these two mutations into 12× mutant were not compatible, and the NADPH regeneration reaction of the derivative mutant (12×-A176R) showed catalytic efficiency of approximately 15 μmol^–1^ min^–1^ and was examined only at a lower temperature (∼25°C) (Johannes et al., 2007).

Thermostability is one of the important properties for industrial biocatalysts because it provides robustness and expands the range of operating temperature. Previously, we isolated RsPtxD from a soil bacterium *Ralstonia* sp. 4506, which shows a 3,450-fold longer half-life at 45°C (80.5 h) than that of PsePtxD. RsPtxD has a 54% amino acid sequence identity to PsePtxD and 7-times higher catalytic efficiency than PsePtxD (Hirota et al., 2012). However, RsPtxD poorly shows its activity on NADP (Hirota et al., 2012), limits its use as an NADPH regeneration system. In this study, we created RsPtxD mutants with high thermostability and improved substrate specificity toward NADP. Our biochemical analysis showed that the NADP binding affinity of RsPtxD mutants was drastically increased, which contributed to not only increased heat stability, but also increased tolerance toward organic solvents. The applicability of RsPtxD mutant as an NADPH regeneration system was evaluated during the conversion of 3-dehydroshikimate (3-DHS) to shikimic acid (SA) at 45°C by a thermophilic shikimate dehydrogenase from *T. thermophilus* HB8.

## Materials and Methods

### Site-Directed Mutagenesis of RsPtxD

The expression plasmids of the His-tagged RsPtxD mutants were constructed from *RsptxD*/pET21b plasmid (Hirota et al., 2012) using a PrimeSTAR Mutagenesis Basal Kit (TaKaRa Bio, Shiga, Japan) with specific primer pairs (Supplementary Table 1). PCR reaction was conducted according to the manufacturer’s instruction. All mutant plasmids were sequenced to verify the mutation of the sequences.

### Protein Expression and Purification

For the expression of mutant RsPtxD proteins, *Escherichia coli* Rosetta 2 (DE3) pLysS (Novagen) transformed with the *RsptxD* mutant plasmids were cultured overnight in the 2×YT liquid medium (Sambrook et al., 1989) at 37°C. Then, 1% of this culture was inoculated in 50 mL of fresh medium and incubated at 37°C until cells reached an optical density at 600 nm (OD_600_) of approximately 0.5. Protein expression was induced with 0.2 mM of isopropyl β-D-thiogalactopyranoside (IPTG) at 28°C for 6 h. The cells were harvested by centrifugation and resuspended in 5 mL of 20 mM Tris–HCl (pH 7.4), 50 mM NaCl, and 20% glycerol. The cell pellets were lysed using sonication on ice for 4 min with a pulse sequence of 1 s on and 2 s off at 20% amplitude level. The crude extracts were then centrifuged at 20,000 ×*g* for 30 min at 4°C to remove the cell debris. The supernatant was filtered through a 0.45-μm filter and loaded onto a 1 mL HisTrap FF column (GE Healthcare UK Ltd., Little Chalfont, United Kingdom) equilibrated in buffer A [20 mM Tris–HCl (pH 7.4), 50 mM NaCl, and 20% glycerol]. After the binding of the Histidine-tagged recombinant proteins, the column was washed with 10 mL of buffer A, and eluted with a linear 10 mL gradient of 0–0.5 M imidazole in buffer A. The eluted protein fractions were pooled, and the buffer was substituted to 50 mM Tris–HCl (pH 7.4) and 15% glycerol by ultrafiltration using Amicon Ultra centrifugal filtration devices (10 kDa molecular mass cutoff) (Merck Millipore, Darmstadt, Germany).

For the expression of shikimate dehydrogenase (SDH), *E. coli* Rosetta 2 (DE3) pLysS was transformed with plasmid pET-11a carrying a gene encoding SDH from *T. thermophilus* HB8 genome (Yokoyama et al., 2000). The protein expression was induced as described above except the concentration of IPTG in the culture was 0.4 mM. The cells were harvested and suspended in 20 mM Tris–HCl (pH 8.0) containing 50 mM NaCl and 20% glycerol, disrupted as described above, and heated at 80°C for 10 min to inactivate the *E. coli* proteins. Subsequently, the cell debris and denatured proteins were removed by centrifugation at 20,000 ×*g* for 30 min. The filtered crude extract was applied onto a 5 mL HiTrap SP FF column (GE Healthcare UK Ltd., Little Chalfont, United Kingdom) equilibrated with a buffer containing 50 mM HEPES (pH 7.0) and 15% glycerol. The SDH protein was eluted with a linear gradient of 0–1.0 M NaCl in the same buffer. The fractions containing SDH were collected and the solution was substituted as described above.

### Enzyme Kinetics

The assays were performed in solutions containing phosphite, NAD(P), 5 μg/mL PtxD protein, and 100 mM morpholinepropanesulfonic acid (MOPS, pH 7.3). The kinetic parameters for one substrate were determined by varying its concentration, whereas the other substrate was added at saturated concentrations (1 mM). The initial rates were determined by measuring the increase in absorbance at 340 nm, which corresponds to NAD(P)H production, using a Beckman DU-800 spectrophotometer (Beckman DU 800, Fullerton, CA, United States). The molar extinction coefficient of 6,220 M^–1^ cm^–1^ was used to calculate NAD(P)H concentration. The kinetic parameters, *K*_M_ and *k*_cat_, for NAD(P) and phosphite were determined using the Lineweaver–Burk plots.

### Optimal Temperature for RsPtxD Proteins

The optimal temperature was determined by incubating the reaction mixture at varying temperatures. The activities of the RsPtxD mutants were measured in 20 mM MOPS (pH 7.3) in the presence of 0.5 mM NAD(P) and 2.0 μg/mL of RsPtxD proteins in a reaction volume of 0.5 mL. The reaction was initiated after adding phosphite at a final concentration of 1 mM into the mixture, and NAD(P)H production was monitored at absorbance 340 nm for 30 min.

### Thermostability and Tolerance to Organic Solvents of RsPtxD Mutants

The thermostability assay was performed according to Woodyer et al. (2003) with minor modifications. To examine the effects of NAD(P) binding with RsPtxD mutants on thermostability, a 0.2 mL solution containing 0.2 μg/μL of the RsPtxD mutant in 50 mM MOPS (pH 7.3) was preincubated on ice with or without 0.5 mM NAD(P) for 5 min and incubated at 45°C. A 10 μL aliquot was taken at different times, and the residual activity was measured in the solution containing 0.5 mM NAD(P), 1.0 mM phosphite, and 20 mM MOPS (pH 7.3) at 37°C in a 1.0 mL reaction volume. To investigate the organic solvent tolerance of RsPtxD mutants, a 0.2 mL solution containing 0.2 μg/μL of the RsPtxD mutant in 50 mM MOPS (pH 7.3) was preincubated with or without NAD(P) for 5 min then incubated with different concentrations of *N,N*-dimethylformamide (DMF) at room temperature (25°C) for 6 h. The residual PtxD activity was assayed as described above.

### Optimal pH for RsPtxD Proteins

The activity of the RsPtxD mutants in the pH range of 6.2–9.2 was determined using buffers 2-morpholinoethanesulfonic acid (MES, pH 6.2), MOPS (pH 7.3), Tris (pH 8.0), and N-cyclohexyl-2-aminoethanesulfonic acid (CHES, pH 9.2) at a final concentration of 20 mM. Each reaction was performed in the presence of 0.5 mM NAD(P), 1.0 mM phosphite, and 2.0 μg/mL of protein at 37°C for 10 min. The NAD(P)H production was measured as described above.

### SA Production and Detection

3-dehydroshikimate is converted to SA by SDH with the NADPH regeneration system using RsPtxD. The coupling reaction of SDH and RsPtxD was performed in a solution containing 3-DHS, NADP, phosphite, SDH, and RsPtxD in 100 mM HEPES (pH 8.0). The reaction solutions with the final volume of 500 μL were incubated at 45°C. Fifty-microliter aliquots were periodically removed from the reaction solution and mixed with an equal volume of 20 mM glycine buffer (pH 2.0) to terminate the reaction. The conversion of 3-DHS to SA was monitored with an HPLC system (Jasco, Tokyo, Japan) equipped with a UV detector and column TSKgel ODS-80T_M_ (250 mm × 4.6 mm internal diameter, and particle size; 5 μm, Tosoh). An isocratic mixture of water/0.1 M H_3_PO_4_ buffer (95:5 v/v) at a flow rate of 0.5 mL min^–1^ was used to separate SA and 3-DHS, with concentrations measured spectrophotometrically at 230 nm. In this system, the retention times for SA and 3-DHS were detected at approximately 9.4 and 13.8 min, respectively.

### Statistical Analysis

All experiments, except the data presented in Figure 3B, were performed in triplicates under the same conditions to prove the reproducibility of experimental results. The results shown are the average values with a standard deviation. The data presented in Figure 3B was the representative of two independent experiments, with essentially the same results.

## Results

### Creation of RsPtxD Mutants

**FIGURE 1:**
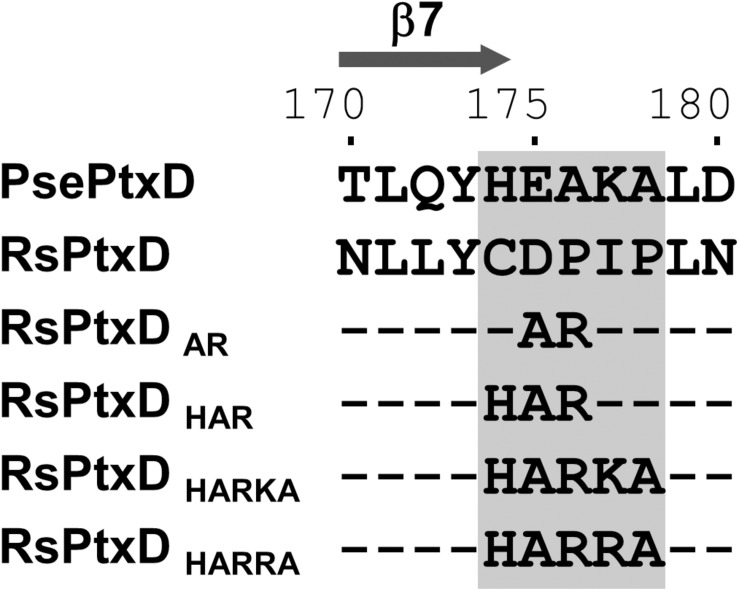
The alignment of the amino acid sequences of PsePtxD, RsPtxD, and RsPtxD mutants. The mutations were introduced at the C-terminus region of the β7-strand in the Rossmann-fold domain (gray rectangle). The comparison of the entire sequences of PsePtxD and RsPtxD is presented in Supplementary Figure 1.

In NAD(P)-dependent dehydrogenases, nicotinamide cofactors are recruited to a nucleotide-binding motif Rossmann fold comprising two α-helices and three β-strands. The specificity of a nicotinamide cofactor to dehydrogenase is influenced by the amino acids after the second β-strand because this position engages with the 2′-hydroxyl of adenine ribose where the phosphate group is present in NADP (Carugo and Argos, 1997). Thus, the electrostatic status of the amino acid side chains in this position usually affects ligand binding, thus is a determinant of nicotinamide substrate specificity (Petschacher et al., 2014). Previously, the replacements of the amino acids at this position with basic amino acids in several NAD-dependent dehydrogenases resulted in increased affinity toward NADP, whereas replacing with acidic amino acids resulted in increased affinity for NAD (Chánique and Parra, 2018). The sequence alignment of RsPtxD and PsePtxD showed that the amino acids located at the C-terminus of the second β-strand (denoted as β7 in Figure 1 and Supplementary Figure 1) in the Rossmann-fold domain of RsPtxD were acidic aspartate at 175 and neutral proline at 176 (Figure 1), possibly causing electrostatic repulsion against 2′-phosphate of NADP. Therefore, we replaced them with alanine and arginine, similar to that in a previous study (Woodyer et al., 2003). We also mutated the adjacent three amino acids (Cys174, Ile177, and Pro178), which are also considered to affect the nicotinamide cofactor binding. Thus, using site-directed mutagenesis, we created four mutants with replaced amino acids, RsPtxD_AR_ (D175A/P176R), RsPtxD_HAR_ (C174H/D175A/P176R), RsPtxD_HARKA_ (C174H/D175A/P176R/I177K/P178A), and RsPtxD_HARRA_ (C174H/D175A/P176R/I177R/P178A) (Figure 1). All the mutant proteins were expressed in *E. coli* and purified using a nickel-affinity column. SDS-PAGE analysis of each purified protein confirmed the enzyme’s homogeneity with a molecular mass of 40.1 kDa (Supplementary Figure 2).

### Kinetic Analysis of RsPtxD Mutants

The kinetic values of the RsPtxD mutants were determined at the conditions of either NAD(P) or phosphite was saturated. The wild-type RsPtxD exhibited the *K*_M_ for NAD (*K*_M_^NAD^) and NADP (*K*_M_^NADP^) of 7.7 ± 2.3 μM and 418 ± 38.7 μM, respectively, confirming its strong preference for NAD and poor binding with NADP as a cofactor (Table 1). As expected, the introduction of the D175A/P176R mutations to RsPtxD (RsPtxD_AR_) significantly reduced *K*_M_^NADP^ to 56 ± 5.1 μM, indicating that RsPtxD_AR_ mutant increased affinity for NADP compared with the wild-type enzyme. This improvement in affinity for NADP is likely owing to the introduction of the non-polar alanine at residue 175, which neutralizes the negative charge of the aspartate residue, and the basic arginine at position 176, creating a positively charged region that can accept the 2′-phosphate group of NADP. However, the catalytic efficiency (*k*_cat_/*K*_M_)^NADP^ of RsPtxD_AR_ was approximately 20-fold lower than that of a PsePtxD mutant in which the same amino acid replacements were introduced (PsePtxD_E__175__A/A__176__R_) (Table 1 and Supplementary Table 2). Comparison of their amino acid sequences revealed that the basic amino acids histidine and lysine were originally present at 174 and 177 in PsePtxD, respectively, whereas the neutral amino acids cysteine and isoleucine were present at the corresponding positions of RsPtxD (Figure 1). Therefore, we speculated that the adjacent residues Cys174, Ile177, and Pro178 were responsible for the high catalytic efficiency of PsePtxD_E__175__A/A__176__R_ with NADP. Indeed, by introducing the C174H mutation to RsPtxD_AR_ (RsPtxD_HAR_), *K*_M_^NADP^ was reduced by 24 ± 5.2 μM with a slightly increased *k*_cat_. Furthermore, RsPtxD_HARKA_ showed *K*_M_^NADP^ values of 4.4 ± 0.9 μM, indicating that the synergetic effect in reducing *K*_M_^NADP^ can be available by introducing I177K and P178A mutations to RsPtxD_HAR_. Based on this result, we replaced isoleucine at 177 with the more basic arginine residue instead of lysine. The resultant mutant, RsPtxD_HARRA_, exhibited *K*_M_^NADP^ value of 2.9 ± 0.5 μM with the catalytic efficiency (*k*_cat_/*K*_M_)^NADP^ of 44.1 μM^–1^ min^–1^, the best performance among the created mutants.

**TABLE 1:** The kinetic parameters of wild-type RsPtxD and its mutants^*a*^.

	NAD				NADP				CSR^*b*^	RCE^*c*^
Enzyme	*K*_M_ (μM)	*k*_cat_ (min^–1^)	*k*_cat_/*K*_M_ (μM^–1^ min^–1^)	*K*_M_ (μM, Pt)	*K*_M_ (μM)	*k*_cat_ (min^–1^)	*k*_cat_/*K*_M_ (μM^–1^ min^–1^)	*K*_M_ (μM, Pt)		
RsPtxD	7.7 ± 2.3	127 ± 16.8	16.6	37.0 ± 7.0	418 ± 38.7	16.0 ± 6.3	4.0 × 10^–2^	338 ± 41.0	2.3 × 10^–3^	
RsPtxD_AR_	412 ± 21.5	115 ± 13.0	0.3	452 ± 16.1	56 ± 5.1	91.1 ± 3.9	1.6	263 ± 16.4	6.0	0.1
RsPtxD_HAR_	154 ± 14.7	155 ± 2.3	1.0	419 ± 89.9	24 ± 5.2	118 ± 6.8	4.9	208 ± 6.2	5.0	0.3
RsPtxD_HARKA_	130 ± 10.6	154 ± 7.5	1.2	388 ± 46.6	4.4 ± 0.9	129 ± 12.1	29.6	27 ± 9.0	25.0	1.8
RsPtxD_HARRA_	112 ± 1.3	148 ± 14.8	1.3	414 ± 45.8	2.9 ± 0.5	128 ± 16.6	44.1	38 ± 8.5	34.0	2.7

*^a^All assays were performed three times at 37°C, pH 7.3, in 100 mM MOPS.^b^Cofactor Specificity Ratio = (k_cat_/K_M_)^NADP^/(k_cat_/K_M_)^NAD^.^c^Relative Catalytic Efficiency = (k_cat_/K_M_)^NADP^_mut_/(k_cat_/K_M_)^NAD^_wt_.*

### Optimal Temperature, Thermostability, and Organic Solvent Tolerance of RsPtxD Mutants

**FIGURE 2:**
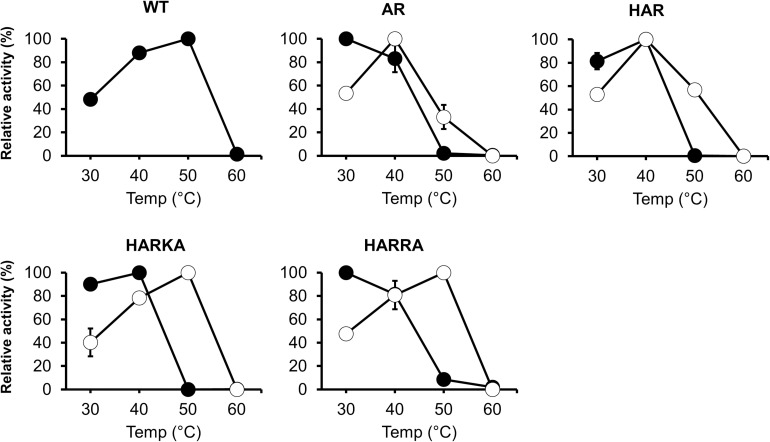
The temperature dependence profiles of the wild-type and mutant RsPtxD proteins. Each protein’s activity was measured using NAD (filled circles) or NADP (open circles) as a nicotinamide cofactor. The data are shown as means ± standard deviation obtained from three independent experiments. WT, wild-type; AR, RsPtxD_AR_; HAR, RsPtxD_HAR_; HARKA, RsPtxD_HARKA_; HARRA, RsPtxD_HARRA_.

**FIGURE 3:**
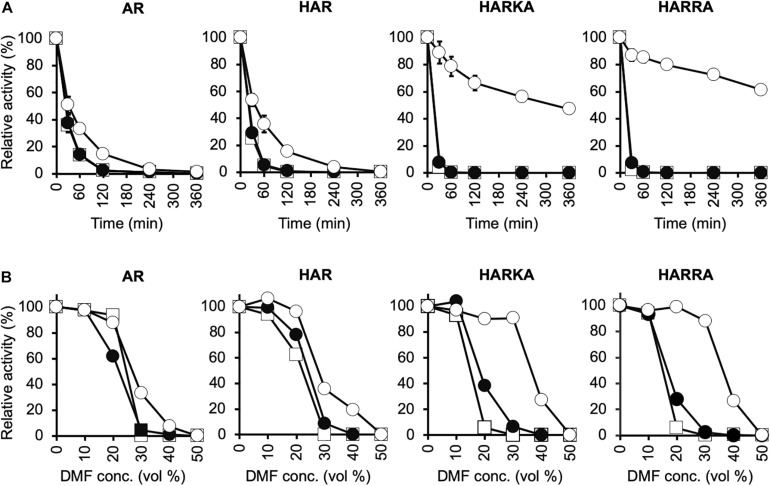
Characterization of the thermal stability and tolerance to organic solvents for RsPtxD variants. **(A)** Thermal inactivation of RsPtxD mutants at 45°C. Data are presented as the mean ± standard deviation from three experiments. **(B)** Stabilities of RsPtxD mutants in DMF. Prior to the assay reaction, RsPtxD mutants were incubated in the 50 mM MOPS (pH7.3) containing 0.5 mM NAD (filled circles), 0.5 mM NADP (open circles), or without cofactor (squares). The reactions were performed by using 0.5 mM NADP as a nicotinamide substrate. The data are representative of two independent experiments. AR, RsPtxD_AR_; HAR, RsPtxD^HAR^; HARKA, RsPtxD^HARKA^; HARRA, RsPtxD_HARRA_.

To investigate the optimum temperature of the RsPtxD mutants, we performed enzyme assays at different temperatures ranging from 30 to 60°C (Figure 2). The wild-type RsPtxD displayed the highest activity at 50°C with NAD as a cofactor. The optimum temperature for RsPtxD_AR_ and RsPtxD_HAR_ was found to be 30 to 40°C with NAD and 40°C with NADP, suggesting that D175A/P176R mutations lowered the optimal temperature, although these mutations increased the affinity toward NADP (Table 1). However, interestingly, the additional mutations of I177K/P178A or I177R/P178A to RsPtxD_HAR_ restored the optimal temperature with NADP to 50°C (Figure 2). Next, we assayed the thermostability of the RsPtxD mutants at 45°C with or without cofactor binding. The thermostabilities of all RsPtxD mutants decreased substantially in the absence of a cofactor (Figure 3A). However, preincubating RsPtxD_AR_ or RsPtxD_HAR_ with 0.5 mM NADP but not 0.5 mM NAD, slightly increased their thermostability (Figure 3A). Meanwhile, preincubating with 0.5 mM NADP but not 0.5 mM NAD significantly increased RsPtxD_HARKA_ and RsPtxD_HARRA_ thermostabilities over 6 h (Figure 3A).

Because thermotolerant enzymes often show resistance to organic solvents, we expected that the protective effect of ligand binding on the RsPtxD mutants would also increase their stability in organic solvents. When RsPtxD proteins were preincubated with or without NAD before incubation at different concentrations of DMF, all the mutants lost their activities at 30% DMF after 6 h (Figure 3B). However, when the RsPtxD mutants were preincubated with NADP, RsPtxD_HARKA_ and RsPtxD_HARRA_ showed approximately 90% of the residual activities in 30% DMF. The protective effect of NADP-binding was not seen with RsPtxD_AR_ and RsPtxD_HAR_ mutants. Thus, these data strongly indicate that strong cofactor binding increases the tolerance for organic solvents of the mutant proteins, i.e., RsPtxD_HARKA_ and RsPtxD_HARRA_.

### The Optimal pH of RsPtxD Mutants

**FIGURE 4:**
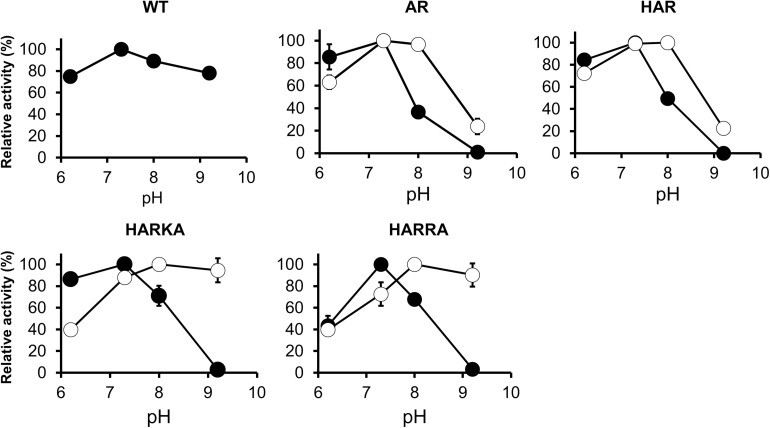
pH dependence profiles of the wild-type and mutant RsPtxD proteins. RsPtxD activity was measured using NAD (filled circles) or NADP (open circles) as substrates. The data are shown as means ± standard deviation obtained from three independent experiments. WT, wild-type; AR, RsPtxD_AR_; HAR, RsPtxD_HAR_; HARKA, RsPtxD_HARKA_; HARRA, RsPtxD_HARRA_.

The activity of the RsPtxD mutants was also examined within the pH range of 6.2–9.2 (Figure 4). When NAD was used as a cofactor, all the RsPtxD mutants demonstrated the highest activity around pH 7.3. Their activities decreased with increasing pH and vanished at pH 9.2, whereas the wild-type RsPtxD retained more than 80% of its activity (Figure 4). When NADP was used as a cofactor, the activities of RsPtxD_AR_ and RsPtxD_HAR_ were optimal between pH 7.3 and 8.0; however, their activities decreased to approximately 20% at pH 9.2 (Figure 4). In contrast, the optimal pH shifted significantly to a higher range for RsPtxD_HARKA_ and RsPtxD_HARRA_ (Figure 4). These results indicate that RsPtxD_HARKA_ and RsPtxD_HARRA_ have a higher affinity for NADP coupling with increased activity at higher pH values.

### SA Production Using RsPtxD as an NADPH Generation System

**FIGURE 5:**
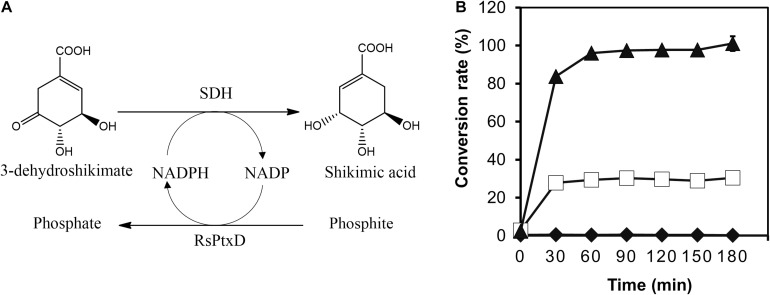
Shikimic acid (SA) production by shikimate dehydrogenase with NADPH regeneration by RsPtxD. **(A)** A schematic of the coupled reactions of SA production by shikimate dehydrogenase (SDH) and NADPH regeneration by RsPtxD. **(B)** The batch production of SA from 3-dehydroshikimate by SDH with NADPH regeneration by wild-type RsPtxD (diamonds), RsPtxD_AR_ (squares), and RsPtxD_HARRA_ (triangles). The reaction solutions contained 20 mM phosphite, 8 mM 3-DHS, 0.1 mM NADP, 0.17 U/mL of SDH, and 20 μg/mL of RsPtxD proteins.

To demonstrate the effect of the RsPtxD mutants as a cofactor regeneration system, the NADPH regeneration reaction of RsPtxD was coupled with the SA production by a thermostable SDH from *T. thermophilus* HB8 (Figure 5A). SDH catalyzes the reduction of 3-DHS to SA, a precursor for the antiviral drug oseltamivir (Tamiflu^®^), using NADPH as a cofactor (Ghosh et al., 2016). In the coupling reaction, 8 mM of 3-DHS was used as the substrate in the presence of 0.1 mM NADP. The wild-type RsPtxD could not support SA production at 45°C, suggesting that it did not regenerate NADPH (Figure 5B). RsPtxD_AR_ supported approximately 28% of the substrate conversion; however, the reaction ceased after 30 min, probably due to heat inactivation. In contrast, RsPtxD_HARRA_ supported almost the full conversion of 3-DHS to SA for 90 min, suggesting that it regenerated NADPH without losing its activity at 45°C. The resultant SA productivity was approximately 22 g L^–1^d^–1^ with a total turnover number (TTN) of 78. A coupling reaction with increasing amounts of substrate, 100 mM 3-DHS, could achieve 61 g L^–1^d^–1^ productivity with a TTN of 860 for NADPH (Supplementary Figure 3).

## Discussion

### The Increased Affinity of RsPtxD Mutants for NADP

Switching nicotinamide cofactor specificity of dehydrogenases is of great interest in the development of industrial biocatalysts. Considering the high cost of NADPH, tremendous efforts have been conducted to alter the cofactor specificity of NADH-regenerative enzymes from NAD to NADP. In the present study, we changed the cofactor specificity of a thermostable PtxD isolated from the soil bacterium *Ralstonia* sp. 4506 (RsPtxD) by mutating five residues, Cys174, Asp175, Pro176, Ile177, and Pro178, located at the C-terminus of the β7-strand region, which interacted with the adenosine ribose moiety of a nicotinamide cofactor. Mutant RsPtxD enzymes enhanced specificity toward NADP and catalytic turnover with robustness against high temperatures and organic solvents. Previously, increased cofactor specificity of PsePtxD to NADP was demonstrated by replacing Glu175 and Ala176 with Ala and Arg, respectively (PsePtxD_E__175__A/A__176__R_) (Woodyer et al., 2003). We found that changes in corresponding amino acids in RsPtxD (RsPtxD_AR_) significantly reduced the *K*_M_ for NADP, confirming that amino acid replacements at this region, especially from acidic to basic, was critical in altering cofactor specificity. However, the *K*_M_ of RsPtxD_AR_ for NADP was still higher than PsePtxD_E__175__A/A__176__R_ (Table 1 and Supplementary Table 2). The additional mutations at Cys174His, Ile177Lys, and Pro178Ala in RsPtxD_AR_, resulted in RsPtxD_HARKA_, greatly reducing the *K*_M_ for NADP, and increasing the catalytic efficiency. The replacement of Ile177 with Arg generated the ideal mutant, RsPtxD_HARRA_, which showed the highest catalytic performance of all mutants. These results demonstrated that not only were Asp175 and Pro176, but also adjacent amino acids of RsPtxD, crucial in recognizing nicotinamide cofactors. A molecular modeling analysis based on the RsPtxD structure (Protein Data Bank ID 6IH3, Liu et al., 2019) suggested that the replacement of Cys174 with His stabilized the binding of nicotinamide cofactors in RsPtxD_AR_ by creating a more compact binding pocket where the adenine moiety of NAD(P) was accommodated (Supplementary Figure 4). The reduction in the *K*_M_s of RsPtxD_HAR_ for both NAD and NADP compared with those of RsPtxD_AR_ is consistent with this modeling (Table 1). The substitution of Ile177 with Lys further reduced the *K*_M_ of RsPtxD_HAR_, possibly by creating a positively charged surface at the entrance of the NAD(P)-binding pocket and promoting NADP loading with proper direction of the 2′-phosphate group into the binding site. Replacing Ile177 with the more basic arginine, as in the case of RsPtxD_HARRA_, contributed to increased hydrogen-bonding interactions and ionic interactions with NADP (Supplementary Figure 4). Protein-ligand affinity is predicted by GBVI/WSA dG, which determines the binding free energy of the ligand from a given pose (Wojciechowski and Lesyng, 2004; Labute, 2008). Molecular modeling analysis of the four RsPtxD mutants using this calculation program yielded the highest affinity in RsPtxD_HARRA_-NADP complex (-9.66 kcal/mol) out of the four analyzed complexes (Supplementary Figure 4).

### The Optimal pH Shift of RsPtxD Mutants

A strong shift to higher pH for maximum activity was observed for RsPtxD_HARKA_ and RsPtxD_HARRA_ mutants, where a positively charged residue, Ile177Lys and Ile177Arg, was introduced at the NADP-binding region, respectively (Figure 4). Similar results by Zhang et al. (1999) reported the activity of wild-type alcohol dehydrogenase from *Vibrio harveyi* (Vh-ALDH) was decreased at a higher pH (pH 8–9), whereas for its mutants (Thr175Asp and Thr175Glu), whose cofactor specificity was changed from NADP to NAD, were all increased in the same pH range. Curiously, these authors observed that the *K*_M_ of the mutants for NAD at pH 9.0 increased approximately 10-fold when compared with values at pH 8.0. Although the reason for this mechanism was unclear, they concluded that the increase in activity from pH 8.0 to 9.0 for these mutants primarily represented an increase in *K*_cat_. Therefore, we measured the activity of the RsPtxD_AR_ mutant at increased NADP concentrations (1.0 mM and 1.5 mM). Consequently, the optimal pH of RsPtxD_AR_ shifted from 7.3 to 8.0, and the relative activities at pH 9.2 increased to approximately 55% and 70% of the maximum activity in the reactions containing 1.0 mM and 1.5 mM NADP, respectively (Supplementary Figure 5A). These results strongly suggested that the reduced relative activity of RsPtxD_AR_ at pH 9.2 was likely due to the lowered *K*_M_, which can be compensated by the increased NADP concentration. The increased affinities of RsPtxD_HARKA_ and RsPtxD_HARRA_ to NADP described earlier may contribute to the high relative activity at high pH (∼90% of the maximum activity at pH 9.2, Figure 4).

### Increased Affinity of RsPtxD Mutants for NADP Contributes to Increased Thermostability and Solvent Stability

Thermostability assays showed that NADP binding protected RsPtxD_HARKA_ and RsPtxD_HARRA_, but not RsPtxD_AR_ and RsPtxD_HAR_, from heat treatment at 45°C. By preincubating RsPtxD_HARRA_ with 0.5 mM NADP, it retained approximately 60% of its activity even after heat treatment for 6 h. In contrast, RsPtxD_HARRA_ preincubated with NAD lost its activity within an hour, at the same temperature. We also observed that an increased concentration of NADP (1.5 mM) in the thermal stability assay compensated for the low affinity of RsPtxD_AR_ (Supplementary Figure 5B). These results illustrated a clear correlation between the thermal protection effect and the binding affinity of RsPtxD mutants toward NADP. Similar thermal protection effects of NAD(P) ligand-binding to enzymes were reported in PsePtxD (Woodyer et al., 2003), aldehyde dehydrogenase of *V. harveyi* (Zhang et al., 1999), and a glyceraldehyde-3-phosphate dehydrogenase of *Bacillus stearothermophilus* (Corbier et al., 1990). In all cases, binding with the preferred nicotinamide cofactor affords the enzyme protection against thermal inactivation, whereas another cofactor with lower affinity provides little or no protective effects. As described earlier, RsPtxD_HARRA_ had increased affinity with NADP owing to its increased hydrogen-bonding formation and ionic interactions. Several reports suggested that strong ligand binding increased protein thermostability due to the coupling of the binding with unfolding equilibrium (Fukada et al., 1983; Brandts and Lin, 1990; Shrake and Ross, 1990). Therefore, the increased affinity of RsPtxD_HARRA_ to NADP likely contributed to the increased thermostability of these mutant enzymes. This ligand binding stabilization effect might also be attributed to the tolerance of organic solvents of the NADP-bound RsPtxD_HARRA_. Because conducting industrial enzymatic reactions in organic solvents provides attractive advantages, such as the increased solubility of organic substrates (Doukyu and Ogino, 2010), the increased organic solvent stability of RsPtxD_HARRA_ together with increased thermostability further expanded the potential applications of PtxD enzymes.

Relyea and van der Donk (2005) proposed that the PtxD reaction is an ordered bi-bi mechanism in which the substrates NAD binding first followed by phosphite, while the products phosphate is released first followed by NADH. In this model, NAD binding induces a conformational change in PtxD, permitting phosphite access to the binding pocket. This was consistent with our observations that as the affinity of RsPtxD mutants to NADP increased, their affinity toward phosphite also increased. The reverse effects when NAD was used as a cofactor also supported this model (Table 1). Taken together, increased NADP-binding with RsPtxD_HARRA_ contributed to increased heat stability and solvent stability, as well as the affinity toward phosphite.

### Comparisons With Other Research Studies for Cofactor Switching and Improvements in NADP-Regeneration Enzymes

According to a recent review, which summarizes the studies of changing the cofactor specificity of 52 NAD-dependent enzymes from NAD to NADP, only 30% of the cases resulted in the variants whose catalytic efficiency with NADP was better than the catalytic efficiency of the wild-type enzymes with their natural cofactor, NAD (Relative Catalytic Efficiency, RCE > 1.0) (Chánique and Parra, 2018). In this work, RsPtxD_HARRA_ showed its RCE value of 2.7 (Table 1), meaning that the catalytic efficiency for NADP was significantly superior compared with that of wild-type RsPtxD for NAD. The changes in the degree of preference of the target cofactor in the mutant enzyme is expressed as coenzyme specificity ratio (CSR). The CSRs of RsPtxD and RsPtxD_HARRA_ were 2.3 × 10^–3^ and 34.0, respectively, suggesting alterations of the amino acid residues contacting the 2′-position of NAD and the residues in the adjacent positions were both important in changing the preference for target cofactors.

As stated in the introduction, FDH is one of the popular NADPH regenerative enzymes currently available (Serov et al., 2002). To date, there have been several attempts to increase the catalytic efficiency of FDH for NADP; two of the recent attempts have been successful. Jiang et al. (2020) increased the catalytic efficiency of the FDH from *Burkholderia stabilis* 15516 to approximately 5.9 μM^–1^ min^–1^ by structural modeling analysis. Calzadiaz-Ramirez et al. (2020) performing *in vivo* selection with an *E. coli* host unable to produce NADPH, identified an FDH mutant from *Pseudomonas* sp. 101 (PseFDH-V9) that showed catalytic efficiency at approximately 8.5 μM^–1^ min^–1^, which was the best value among the previously reported FDHs. Although both enzymes show relatively high *K*_cat_ values, their catalytic efficiencies are approximately 5–7 times less than that of RsPtxD_HARRA_. Moreover, FDHs generally require a high substrate concentration owing to their high *K*_M_ for formate (9–31 mM) (Supplementary Table 2). In contrast, RsPtxD_HARRA_ shows low *K*_M_s for both NADP and phosphite, meaning that the amount of required substrates can be reduced without affecting its maximal activity.

### Conclusion

In this study, we developed an NADP-specific RsPtxD mutant, RsPtxD_HARRA_, that showed high catalytic efficiency, thermostability, and organic solvent stability. Our biochemical analyses showed these improved characteristics were due to the high affinity of RsPtxD_HARRA_ for its preferred cofactor, NADP. We also demonstrated the effectiveness of RsPtxD_HARRA_ as an NADPH regeneration enzyme for the chiral production of SA. Considering its high catalytic efficiency and high stability in organic solvent and heat conditions, RsPtxD_HARRA_ provides a promising alternative to the NADPH regeneration system in industrial applications.

## Data Availability Statement

The original contributions presented in the study are included in the article/Supplementary Material, further inquiries can be directed to the corresponding author/s.

## Author Contributions

GA-H and RH performed the experiments and wrote the manuscript. TIk, TIs, HF, and AK performed the data analysis. AK and RH conceived the project. All authors contributed to the article and approved the final version of the manuscript.

## Conflict of Interest

The authors declare that the research was conducted in the absence of any commercial or financial relationships that could be construed as a potential conflict of interest.
