# Supplementary material for: Engineering Cofactor Specificity of a Thermostable Phosphite Dehydrogenase for a Highly Efficient and Robust NADPH Regeneration System
Source: Front Bioeng Biotechnol. 2021 Apr 1;9:647176. doi: 10.3389/fbioe.2021.647176 (PMC8047080; doi:10.3389/fbioe.2021.647176)
Supplement: Supplementary file 1 [file Data_Sheet_1.pdf]

Supplementary Information for

# Engineering Cofactor Specificity of a Thermostable Phosphite Dehydrogenase for a Highly-efficient and Robust NADPH Regeneration System

**Gamal Nasser Abdel-Hady[1,2], Takeshi Ikeda[1,3], Takenori Ishida[1,3], Hisakage Funabashi[1,3] Akio Kuroda[1,3], Ryuichi Hirota[1,3*]**

[1] Department of Molecular Biotechnology, Graduate School of Advanced Sciences of Matter, Hiroshima University, Hiroshima, Japan

[2] Department of Genetics, Faculty of Agriculture, Minia University, Minia, Egypt

[3] Unit of Biotechnology, Division of Biological and Life Sciences, Graduate School of Integrated Sciences for Life, Hiroshima University, Hiroshima, Japan

*** Correspondence :**

Ryuichi Hirota

hirota@hiroshima-u.ac.jp

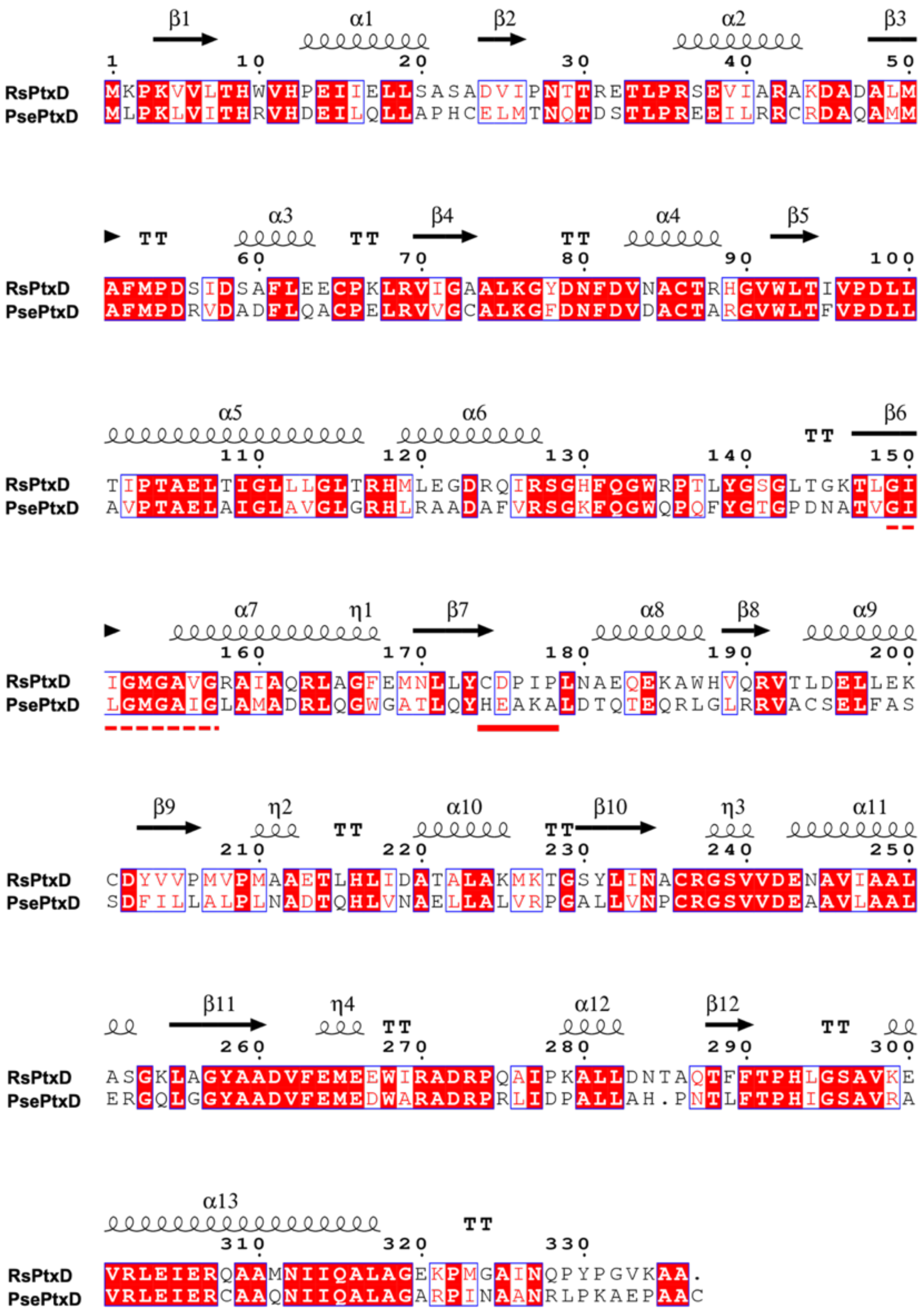


**Supplementary Figure 1.** Sequence alignment of phosphite dehydrogenases from *Ralstonia* sp. 4506 (RsPtxD) and *P. stutzeri* WM88 (PsePtxD). The alignment was generated using ESPrint 3.0 (Robert and Gouet 2014; http://espri pt.ibcp.fr/ESPri pt/cgi-bin/ESPri pt.cgi). The α-helixes (coiled lines) and β-sheets (solid arrows) are shown at the top of the alignment sequence. The GxxGxGxxG motif and the target site for the amino acid substitutions are shown below the alignment by a red dashed line and solid red line, respectively.

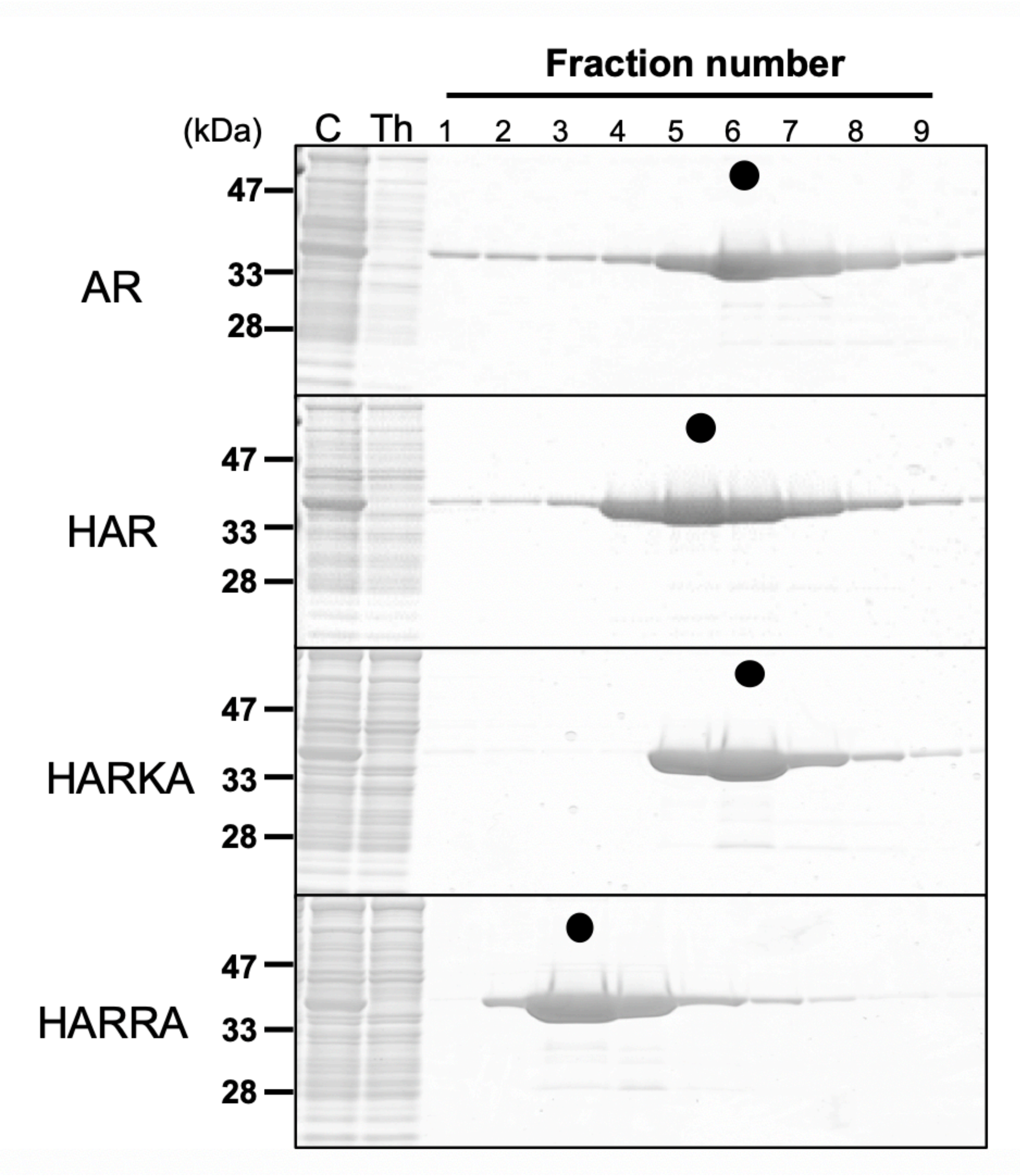


**Supplementary Figure 2.** Purification of the recombinant RsPtxD proteins using a nickel column. Indicated fractions by black dots were collected, buffer exchanged, and concentrated for the analysis. C: crude extract, Th: through fraction. AR, RsPtxD$_{AR}$. HAR, RsPtxD$_{HAR}$. HARKA, RsPtxD$_{HARKA}$. HARRA, RsPtxD$_{HARRA}$.

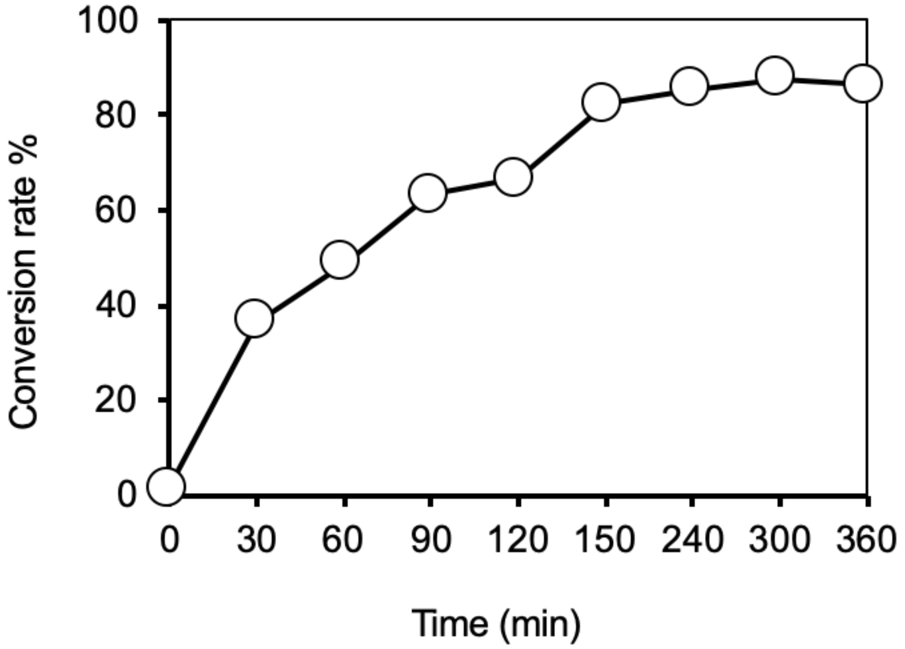


**Supplementary Figure 3.** Batch production of SA from 3-DHS with regeneration of NADPH using RsPtxD$_{HARRA}$ mutant. The reaction mixture contained 100 mM 3-DHS, 0.1 mM NADP, 150 mM phosphite, 40 μg mL$^{-1}$ SDH, and 160 μg mL$^{-1}$ RsPtxD$_{HARRA}$.

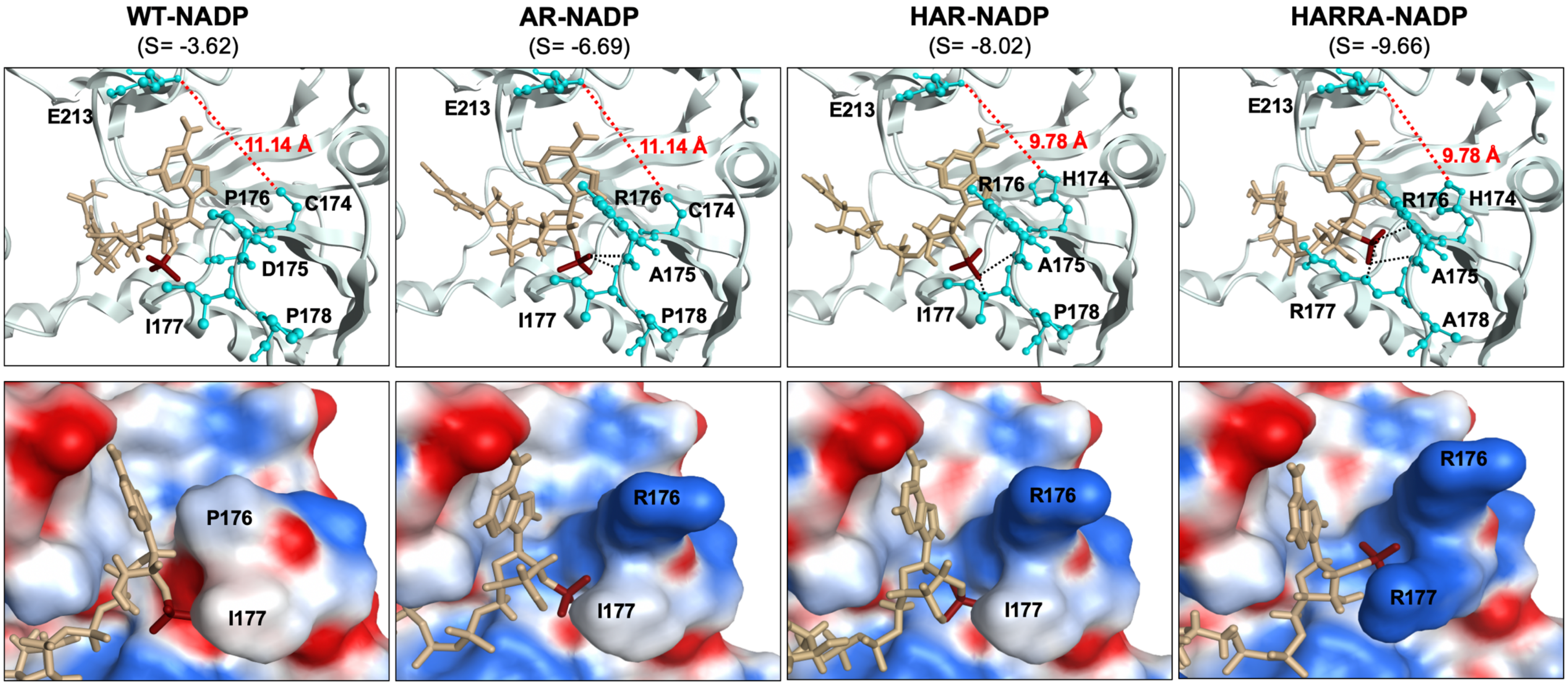


**Supplementary Figure 4.** Proposed model structures of the NAD-binding pocket of RsPtxD (WT), RsPtxD$_{AR}$ (AR), RsPtxD$_{HAR}$ (HAR), and RsPtxD$_{HARRA}$ (HARRA) (Top panels). The side chains of amino acid residues at 174, 175, 176, 177, 178, and 213 are shown in cyan. The protein structures were modeled by the Molecular Operating Environment software (MOE; Chemical Computing Group Inc., Montreal, Canada) using the PDB 6IH3 structure (Liu et al., ACS Catalysis 9: 1893-1887, 2019) as a template. The distance between the residues at 174 and 213 was shown with a red dotted line. The NAD moiety was shown in light brown and the 2'-phosphate group was shown in dark red. Note that the distances between the residues 174 and 213 in RsPtxD$_{HAR}$ and RsPtxD$_{HARRA}$ are shorter than that in RsPtxD and RsPtxD$_{AR}$. Hydrogen bonding between residues and cofactor were shown as black dotted lines. The numbers (S values) in the parenthesis are binding energy (kcal/mol) of NADP and RsPtxDs calculated by the GBVI/WSA dG scoring function of the MOE software. The electrostatic surface potentials of the proposed model structures of the NAD-binding pocket of RsPtxD and its mutants (Bottom panels). Positively charged region is indicated in blue and negatively charged region in red.

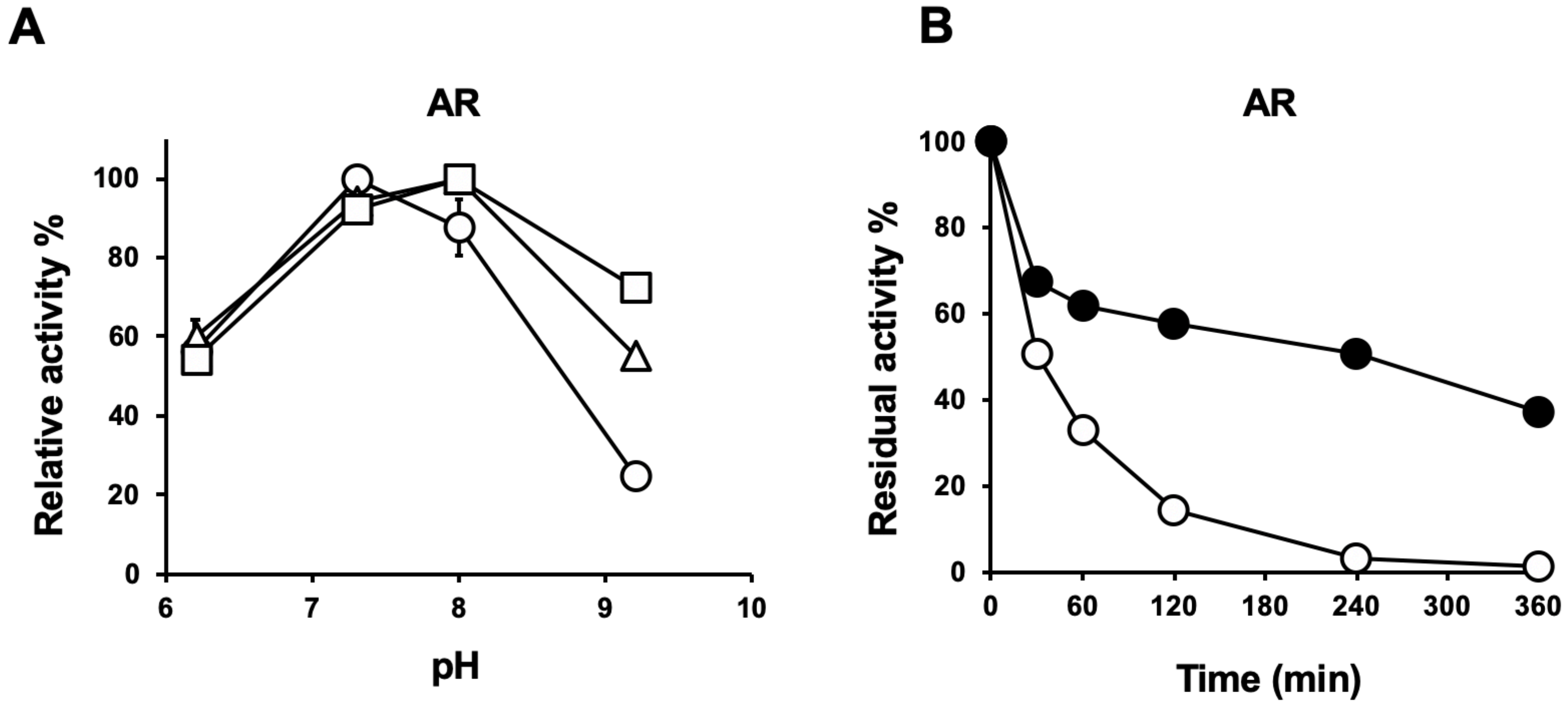


**Supplementary Figure 5.** pH dependent profile (A) and thermal stability (B) of RsPtxD$_{AR}$ mutant (AR). (A) PtxD activity was measured using 0.5 mM (circles), 1.0 mM (triangles), and 1.5 mM (squares) of NADP. The data are shown as means ± standard deviation obtained from three independent experiments. (B) Thermal inactivation of RsPtxD$_{AR}$ was performed with 0.5 mM (open circles) and 1.5 mM (closed circles). The data was representative of two independent experiments, with essentially the same results.

**Supplementary Table 1.** The oligonucleotides and plasmids used in this study

| Primers | Sequence 5'-3'* |
|---|---|
| D175A/P176R_fw | TTGC**GCACGT**ATTCCGCTCAATGCCGAA |
| D175A/P176R_rv | GGAAT**ACGTGC**GCAATACCAGAGATTCA |
| C174H/D175A/P176R _fw | TGTAT**CAC**GCACGTATTCCGCTCAATG |
| C174H/D175A/P176R _rv | ACGTGC**GTG**ATACAAGAGATTCATTTC |
| C174H/D175A/P176R/ I177K/P178A_fw | GCACGT**AAAGCG**CTCAATGCCGAACAAG |
| C174H/D175A/P176R/ I177K/P178A_rv | TGAG**CGCTTT**ACGTGCGTGATACAAGAG |
| C174H/D175A/P176R/ I177R/P178A_fw | GCACGT**CGCGCG**CTCAATGCCGAACAAG |
| C174H/D175A/P176R/ I177R/P178A_rv | TGAG**CGCGCG**ACGTGCGTGATACAAGAG |
| **Plasmids** | **Descriptions** |
| *RsptxD*/pET21b | pET21b (Novagen) containing *RsptxD*<br>(Hirota et al., 2012) |
| *RsptxD*$_{(D175A/P176R)}$/pET21b | pET21b containing a mutant *RsptxD* gene for<br>RsPtxD$_{AR}$ expression |
| *RsptxD*$_{(C174H/D175A/P176R)}$/pET21b | pET21b containing a mutant *RsptxD* gene for<br>RsPtxD$_{HAR}$ expression |
| *RsptxD*$_{(C174H/D175A/P176R/I177K/P178A)}$/pET21b | pET21b containing a mutant *RsptxD* gene for<br>RsPtxD$_{HARKA}$ expression |
| *RsptxD*$_{(C174H/D175A/P176R/I177R/P178A\ )}$/pET21b | pET21b containing a mutant *RsptxD* gene for<br>RsPtxD$_{HARRA}$ expression |
| *sdh*/pET11a | pET11a plasmid containing shikimate<br>dehydrogenase gene from *T. thermophilus*<br>(Yokoyama et al., 2000) |

*Boldface nucleotide sequences indicate mutation positions.

**Supplementary Table 2.** Comparison of kinetic parameters of NADP-dependent enzymes previously reported

| Enzyme | NADP | | | | Assay conditions | References |
|---|---|---|---|---|---|---|
| | $K_M$ (μM, NADP) | $K_{cat}$ ($min^{-1}$) | $K_{cat}/K_M$ ($\mu M^{-1}$ $min^{-1}$) | $K_M$ (μM, Pt or formate) | | |
| $PsePtxD_{E175A/A176R}$ | 3.5 ± 0.5 | 114 ± 33.0 | 32.5 | 21 ± 3.0 | 25 °C, pH 7.25 | Woodyer et al., 2003 |
| $PsePtxD_{12x\text{-}A176R}$ | 5.5 ± 0.7 | 82 ± 4.0 | 14.9 | 36 ± 14.0 | 25 °C, pH 7.25 | Johannes et al., 2007. |
| mut PseFDH | 150 ± 25 | 150 ± 9.0 | 1.0 | 9000 ± 3000 | 30 °C, pH 7.0 | Serov et al., 2002. |
| $BstFDH_{G146M/A287G}$ | 90 ± 0.0 | 529 ± 1.8 | 5.9 | 31700 ± 3700 | 30 °C, pH 7.0 | Jiang et al., 2020. |
| PseFDH-V9 | 26 ± 1.0 | 221 ± 1.8 | 8.5 | 24000 ± 2400 | 30 °C, pH 7.0 | Calzadiaz-Ramirez et al., 2020. |
